# Selection, Identification, and Transcript Expression Analysis of Antioxidant Enzyme Genes in *Neoseiulus barkeri* after Short-Term Heat Stress

**DOI:** 10.3390/antiox12111998

**Published:** 2023-11-13

**Authors:** Tong Zhu, Weizhen Li, He Xue, Shibo Dong, Jianhui Wang, Suqin Shang, Youssef Dewer

**Affiliations:** 1 Biocontrol Engineering Laboratory of Crop Diseases and Pests of Gansu Province, College of Plant Protection, Gansu Agricultural University, Lanzhou 730070, China; judy20200701@126.com (T.Z.); 13934404211@163.com (H.X.); 18894545570@163.com (S.D.); 15095553575@163.com (J.W.); 2 Key Laboratory of Grassland Ecosystem of Ministry of Education, Sino-U.S. Centers for Grazingland Ecosystem Sustainability, College of Grassland Science, Gansu Agricultural University, Lanzhou 730070, China; johnnie0918@126.com; 3 Phytotoxicity Research Department, Central Agricultural Pesticide Laboratory, Agricultural Research Center, 7 Nadi El-Seid Street, Dokki, Giza 12618, Egypt

**Keywords:** *Neoseiulus barkeri*, short-term heat stress, antioxidant enzyme genes, ROS, oxidative stress

## Abstract

Phytoseiid mite *Neoseiulus barkeri* is a crucial biological control agent utilized to control pest mites and many insects in crops all over the world. However, they are vulnerable to multiple environmental pressures, with high-temperature stress being the most significant challenge. Heat stress disrupts the balance of reactive oxygen species (ROS) levels in organisms, resulting in oxidative stress within the body. Antioxidant enzymes play a crucial role in effectively neutralizing and clearing ROS. In this study, comparative transcriptomics and quantitative real-time PCR (qRT-PCR) were employed to assess the impact of short-term heat stress on the transcript expression of antioxidant enzyme genes in *N. barkeri*. We primarily identified four antioxidant enzyme genes (*NbSOD*, *NbPrx*, *NbCAT*, and *NbGPX*) in *N. barkeri* after exposure to short-term heat stress. Then, new data on the expression patterns of these genes were generated. RNA sequencing and bioinformatics analysis revealed that *NbSOD* belongs to the Fe/Mn family of superoxide dismutase (SOD), which was identified as MnSOD. *NbPrx* was classified as a 1-Cys peroxiredoxin of the peroxidase family, whereas *NbCAT* was recognized as a classical catalase, and *NbGPX* was determined as cytoplasmic glutathione peroxidase-1 (GPX1). Transcriptional expression analysis of these four genes was conducted at different high temperatures: 36 °C, 38 °C, and 40 °C for 2, 4, and 6 h. The results also showed that all four genes exhibited significant up-regulation in response to short-term heat stress. Similarly, the highest expression levels for *NbSOD*, *NbPrx*, and *NbCAT* were observed at 40 °C for 4 h. However, *NbGPX* displayed its maximum expression value at 38 °C for 4 h. Overall, the obtained data suggest that short-term heat stress increases levels of ROS generated inside living organisms, which disrupts the oxidative balance and leads to alterations in the expression levels of antioxidant enzyme genes.

## 1. Introduction

*Neoseiulus barkeri* (Acari: Phytoseiidae) is a widely distributed and commercially accessible predator of pest mites and many arthropod pests [1]. It has been reported in Asia, America, Australia, Africa, and Europe [2]. As an ectothermic animal, *N. barkeri* is very susceptible to temperature stress, and at 16 °C, *N. barkeri* will undergo diapause, and only after mating can it lay eggs [3]. With the increase in temperature, its development period and life span shorten [4,5], its development period cannot be completed at 40 °C [5], and the eggs cannot hatch at 42 °C [6]. *N. barkeri* frequently encounters high-temperature stress in both natural environments and facility agriculture. In spite of numerous attempts that have been made in recent years to study the impact of temperature on the developmental rates of arthropods, there is little information related to mite pests at the molecular level [7]. High temperatures can cause a dramatic increase in ROS levels, leading to harmful effects on the population of *N. barkeri* [6,8,9] and resulting in suppressed potential and low control efficiency on small insects and mite pests [10].

Reactive oxygen species (ROS) are reactive compounds derived from oxygen generated in the cellular metabolism of living cells. Normal levels of ROS are essential for cellular signaling networks and physiological functions such as autophagy, pathogen killing, and resolution of inflammation. In contrast, higher levels of ROS lead to irreversible damage of macromolecules involved in various metabolic processes, which eventually harm cellular components and functions [11,12,13]. Several evidences showed that biotic and abiotic stresses may induce a high amount of reactive oxygen species (ROS) concentrations in cells [14]. High temperature is one of the abiotic stresses that cause alterations in insect biology, behavior, morphology, and development, as well as cellular and metabolic processes, which can disturb the delicate equilibrium between ROS production and reduction [15,16,17,18,19]. The primary way in which ROS is eliminated is through the body’s antioxidant defense systems, which include antioxidant enzymes like superoxide dismutases (SOD) and catalase (CAT), as well as direct antioxidants such as vitamin E and glutathione (GSH). These antioxidants are essential for maintaining the balance between oxidation and reduction in the cell and thus play a critical role in reducing ROS levels [20].

The SOD enzyme family is the primary antioxidant defense in organisms. On the one hand, it catalyzes the dismutation of superoxide anion into molecular oxygen and hydrogen peroxide and serves as the first line of enzymatic defense against ROS [21,22]. On the other hand, peroxidase (Prx) and CAT decompose H_2_O_2_ into nontoxic H_2_O and O_2_, causing a low level of free radicals inside the cells that cannot exert a toxic effect. This protects the intact structure and function of cell membranes [23]. Most Glutathione peroxidase (GPX) are selenium-dependent enzymes that catalyze the reduction of H_2_O_2_ and organic hydroperoxides to water and the corresponding alcohols, respectively. They typically use glutathione (GSH) as an electron donor [24]. Based on our previous research, short-term heat stress resulted in increased expression of SOD, CAT, and Prx activities in *N. barkeri*, and the enzyme activity reached its maximum at 40 °C–4 h [25]. A notable rise in antioxidant enzyme activities suggests the presence of oxidative stress and a positive capability to counteract it by eliminating ROS from cells [26,27].

Although the structure and composition of antioxidant enzyme genes are highly conserved, there is limited understanding of the expression mechanism of these genes in invertebrates, particularly mites. In this study, RNA-seq, qRT-PCR, and comprehensive transcriptome analysis were performed on adult females of the predator mite *N. barkeri* subjected to short-term high temperatures (25 °C and 40 °C for 4 h). Firstly, we identified four antioxidant genes (*NbSOD*, *NbPrx*, *NbCAT*, and *NbGPX*), and their differentially expressed genes in response to heat stress were obtained. Second, we conducted bioinformatics analysis on these four genes. Finally, we analyzed the expression patterns of these four antioxidant enzymes after exposure to different temperatures of 36 °C, 38 °C, and 40 °C for 2, 4, and 6 h. These results will improve our understanding of the molecular response mechanism of *N. barkeri* to heat stresses and guide their impacts on the efficacy of the biological control of natural enemies against *N. barkeri*.

## 2. Materials and Methods

### 2.1. Insect Culture

A colony of the predatory mite *N. barkeri* used in this study was maintained in the laboratory for multiple generations (without pesticide exposure) in the Department of Insect Systematics and Biodiversity at the College of Plant Protection, Gansu Agricultural University, China. The susceptible population of *N. barkeri* was kept in plastic boxes measuring 17 cm × 12 cm × 10 cm and fed on a bran/mite mixture (a mixture of *Aleuroglyphus ovatus* Troupeau and bran [6]. The colony was continuously reared in an artificial-control chamber, with a 14/10 h light/dark cycle at temperature (25 °C ± 1 °C) and humidity (80 ± 5%).

### 2.2. RNA-Seq Library Construction and Sequencing

Before RNA-Seq library construction and sequencing, the RNA was first extracted. Six hundred newly emerged adult females (1–2 days old) were collected into a 1.5 mL microtube and placed in the artificial-control chamber under two conditions: 25 °C and 40 °C for 4 h. Samples from 25 °C were identified as the control group, while samples from 40 °C-4 h were identified as the treatment group. After treatments, samples were frozen immediately using liquid nitrogen and stored at −80 °C in a refrigerator for subsequent use. RNA was extracted from each sample using Trizol reagent (Thermo Fisher Scientific, New York, NY, USA), and the quality and quantity of RNA were assessed using an Agilent 2100 Bioanalyzer (Agilent Technologies, California, CA, USA). Only RNA samples with RIN values greater than 8.0 were used for transcriptome sequencing. Each treatment was repeated three times, and 3600 mites were used in the RNA extraction.

After the RNA extraction, 6 RNA-Seq libraries were constructed using 1 μg RNA from each sample, following the protocol described by Chao et al. (2019) [28]. The RNA-Seq libraries were collected and sequenced using the DNBSEQ platform. The transcriptome data was uploaded to the NCBI Sequence Read Archive (SRA) with accession number PRJNA993641.

### 2.3. Transcript Assembly, Gene Annotation, and Gene Expression Level Calculation

Before conducting de novo assembly, raw reads from each library were filtered by removing low-quality reads, reads containing adapters, and reads containing >5% unknown bases. The resulting clean reads were assembled using Trinity 2.2.0 software to produce unigenes [29]. Unigenes were blasted with non-redundant protein sequences in the non-redundant protein sequence database (NR), gene ontology (GO), Kyoto Encyclopedia of genes and genomes (KEGG), eukaryotic orthologous groups (KOG), Swiss Prot and Pfam databases to obtain annotated information using Blastp software (https://blast.ncbi.nlm.nih.gov/Blast.cgi) [30]. Clean reads were aligned to the genome sequence using Bowtie2 (http://bowtie-bio.sourceforge.net/Bowtie2/index.shtml) accessed on 16 April 2023, and gene expression levels were estimated from RNA-Seq data for each sample using the RSEM package.

### 2.4. Selection of Antioxidant Genes

According to the gene expression levels in each sample, the total number of genes identified as differentially expressed (DEG) was detected. Based on gene annotation information obtained from DEG analysis, we screened out the antioxidant enzyme gene regulated by short-term heat stress in *N. barkeri*.

### 2.5. Cloning the CDS of Antioxidant Genes

Primers were designed based on the analysis of transcriptome information of *N. barkeri* (Table S1) to amplify CDS fragments of target genes *NbSOD*, *NbPrx*, *NbCAT*, and *NbGPX*. First-strand cDNA was synthesized using the PrimeScript RT reagent Kit (Takara, Dalian, China), and PCR products were ligated into the pLB-T vector (TIANGEN, China). RNA templates used in cDNA synthesizing were from the control group in Section 2.2. The resulting constructs were transformed into Top10 *Escherichia coli* cultures (TIANGEN, Beijing, China), and positive clones were selected for sequencing using Tsingke Biotech Co., Ltd. (Beijing, China).

### 2.6. Bioinformatic Analysis and Identification of Antioxidant Genes

To identify functional domains in *NbSOD*, *NbPrx*, *NbCAT*, and *NbGPX*, SMART software (http://smart.embl-heidelberg.de/ (accessed on 17 June 2023)) was used. Additionally, WoLF PSORT (https://wolfpsort.hgc.jp// (accessed on 17 June 2023)) was employed for protein subcellular localization prediction. Furthermore, we calculated the molecular weights and theoretical isoelectric points by using the ExPASy ProtParam tool (http://web.expasy.org/protparam// (accessed on 17 June 2023)). The signal peptides were predicted using the SignalP 4.1 Server (http://www.cbs.dtu.dk/services/SignalP// (accessed on 17 June 2023)). To predict the SOD of metal binding sites, we used IntelPro tools (http://www.ebi.ac.uk/interpro/scan.html/ (accessed on 17 June 2023)). Homologous sequences from other invertebrates were obtained from the NCBI database and aligned using GENEDOC v.2.7. (Nicholas, K.B. etc, California, USA, http://www.psc.edu/biomed/genedoc (accessed on 17 June 2023)) To construct a phylogenetic tree, we employed the neighbor-joining algorithm with the bootstrap method (1000 replications) in Molecular Evolutionary Genetics Analysis (MEGA) v.11.0 (Sudhir Kumar, Pennsylvania, https://megasoftware.net/ (accessed on 17 June 2023)), based on the aligned multiple homologous sequences.

### 2.7. Transcript Expression of Antioxidant Genes

In this study, we investigated the expression of antioxidant enzyme genes at the transcriptional level using RT-qPCR. The housekeeping gene β-actin [31] was used as a reference gene for normalization, and the primers used in the study are listed in Table S2. Six hundred newly emerged adult females (1–2 days old) were collected with a little brush into a 1.5 mL microtube and placed in the artificial-control chamber. Samples were treated at different temperatures (36, 38, and 40 °C) for varying durations (2, 4, and 6 h) under controlled humidity (RH 80 ± 5%) and then frozen immediately with liquid nitrogen and stored at −80 °C in a refrigerator after that. Adult females reared at 25 °C were used as negative controls. RNA was extracted from each sample using Trizol reagent (Thermo Fisher Scientific, New York, NY, USA), and the quantity and quality of RNA samples were assessed using a Thermo Scientific NanoDrop^TM^ 2000 UV-VIS Spectrophotometer (Thermo Fisher Scientific, New York, NY, USA). A total of 600 mites were used in RNA extraction in each treatment, and each treatment was replicated three times. Approximately 1 μg RNA from each sample was converted into cDNA using the PrimerScriptTM RT reagent Kit with gDNA Eraser (TaKaRa, Dalian, China).

### 2.8. Statistical Analysis

Quantitative real-time PCR (qRT-PCR) analyses were performed to determine Gene expression using the relative quantification 2^−∆∆CT^ method [32].

## 3. Results

### 3.1. RNA-Seq Data Analysis

Using the DNBSEQ platform, a total of 38.73 Gb clean reads of data were obtained. After assembly and redundancy, 37,379 unigenes were obtained with a total length of 35,395,678 bp, an average length of 946 bp, an N50 of 1955 bp, and a GC content of 49.16% (Table S3). The unigene alignment was then annotated to seven functional databases, resulting in 21,954 (58.73%) NR, 8951 (23.95%) NT, 13,462 (36.01%) Swiss Prot, 12,968 (34.69%) KOG, 16,044 (42.92%) KEGG, 11,039 (29.53%) GO, and 15,042 (40.24%) Pfam unigenes with functional annotations (Table S4). The *N. barkeri* sequences showed the highest similarity with sequences from the predatory mite *Galendromus occidentalis* (81.42%). Other matches included *Tropilaelaps mercedesae* (3.65%), *Varroa jacobsoni* (3.47%), *Varroa destructor* (1.55%), and *Rhagoletis zephyria* (1.16%) (Figure S1).

### 3.2. Identification of Antioxidant Genes

**Figure 1:**
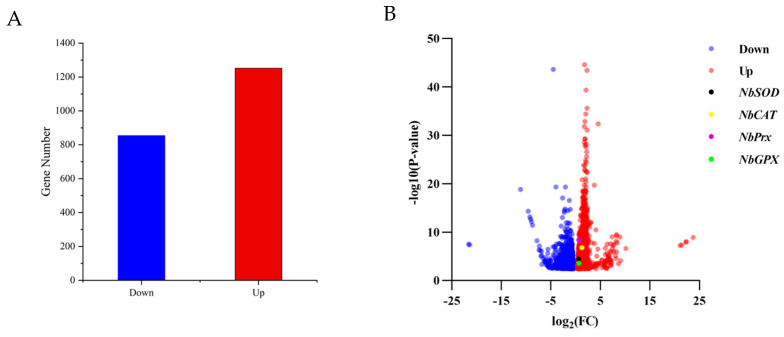
The number of differentially expressed genes and their expression levels in adult female individuals of *N. barkeri* under control (25 °C) vs. treatment (40 °C—4 h) conditions. (**A**). The number of down-regulated and up-regulated genes in differentially expressed genes. If Log2(Treat FPKM/Control FPKM) > 0, it was identified as an up-regulated gene; if Log2(Treat FPKM/Control FPKM) < 0, it was recognized as a down-regulated gene. (**B**). Volcano plot displaying the variability pattern, with red points indicating up-regulated genes, blue points indicating down-regulated genes; *NbSOD*, *NbPrx*, *NbCAT*, and *NbGPX* are shown in black, purple, yellow, and green, respectively. The final result is based on FPKM as the original data, and Log2FC and *p*-value were calculated using the DEseq2 method.

**Figure 2:**
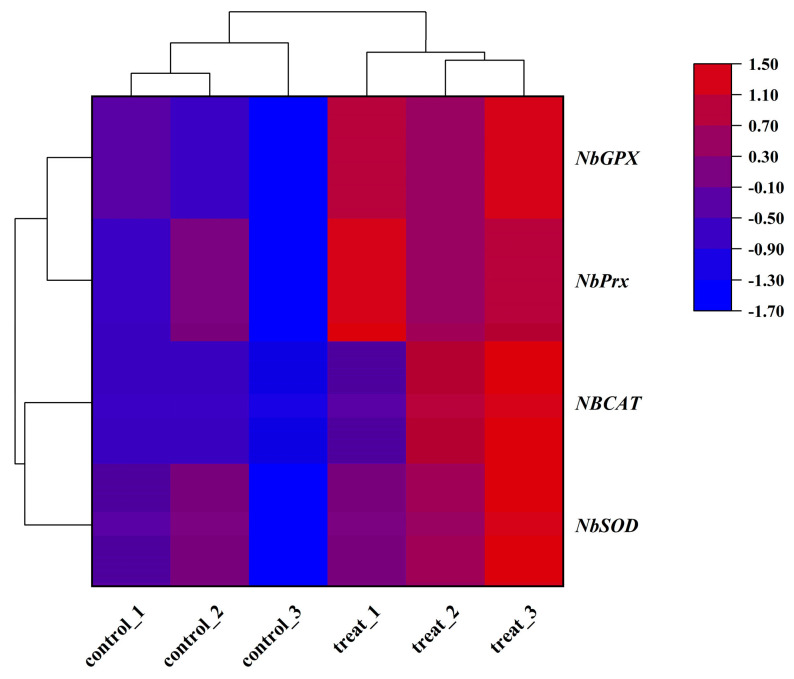
Heat map of the expression levels of four antioxidant genes in adult female *N. barkeri*. The color scale is shown at the upper right, which denotes the FPKM value from lowest (blue) to highest (red).

After a 4 h treatment at 40 °C, *N. barkeri* showed differential expression of 1252 up-regulated and 854 down-regulated transcripts (FDR ≤ 0.001 and log2Ratio ≥ 1) (Figure 1). Among the differentially expressed genes, four antioxidant genes were identified using gene annotation information, which were named *NbSOD*, *NbPrx*, *NbCAT,* and *NbGPX* (gene entry numbers KX505994.1, OR597505, OR597506, and OR597507) (Figure S2). The *NbPrx*, *NbCAT,* and *NbGPX* genes were first identified, except for *NbSOD*. Based on their FPKM values (Figure 2), we calculated the result of Log_2_ (treatFPKM/control FPKM) > 1, so they were identified as up-regulated genes.

### 3.3. Characterization of Antioxidant Genes

**Figure 3:**
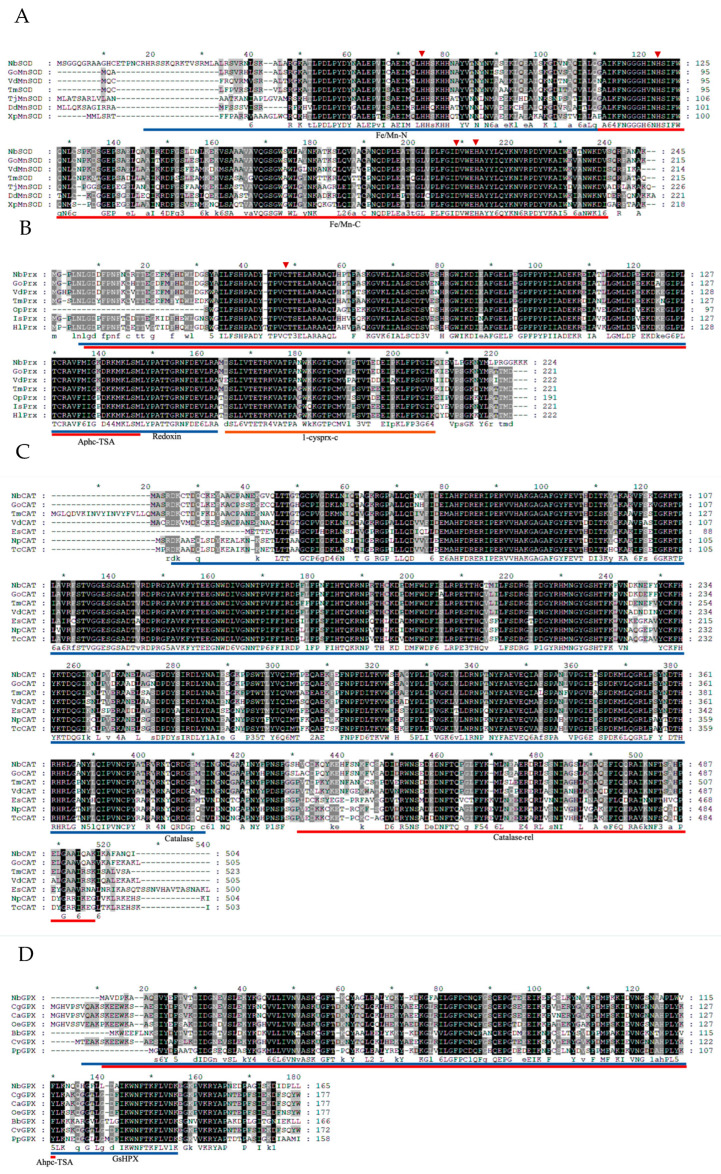
Multiple sequence alignment of four antioxidant proteins ((**A**) *NbSOD*, (**B**) *NbPrx*, (**C**) *NbCAT*, and (**D**) *NbGPX*) with other species. Go: *Galendromus occidentalis*; Vd: *Varroa destructor*; Tm: *Tropilaelaps mercedesae*; Av: *Armadillidium vulgare*; Pc: *Penaeus chinensis*; Cs: *Cryptotermes secundus*; Op: *Ornithodoros parkeri*; Is: *Ixodes scapularis*; Hl: *Haemaphysalis longicornis*; Es: *Eriocheir sinensis*; Np: *Nephila pilipes*; Tc: *Trichonephila clavata*; Cg: *Crassostrea gigas*; Ca: *Crassostra angulata*; Oe: *Ostrea edulis*; Bb: *Burkholderiales bacterium*; Cv: *Crassostrea virginica*; Pp: *Panacagrimonas perspica*; *Conserved domains* are indicated with blue, orange, and red underlines. The red arrow in Figure 3A indicates the manganese ion binding site, as the *NbSOD* was identified as MnSOD. The red arrow in Figure 3B represents the cysteine residual active site at position 46 in *NbPrx*. The black part shows the exact same amino acid sequence in all species, and the gray part shows the similar part in most species.

We successfully cloned and identified four antioxidant enzyme genes named *NbSOD*, *NbPrx*, *NbCAT*, and *NbGPX* from *N. barkeri*. Sequence analysis revealed that *NbSOD*, *NbPrx*, *NbCAT*, and *NbGPX* have ORF lengths of 648, 675, 1515, and 498, encoding 215, 224, 504, and 265 amino acids, respectively. The calculated molecular weights are 23.711, 25.000, 57.212, and 18.547 kDa, with theoretical isoelectric points of 8.85, 5.87, 7.22, and 5.50, respectively (Table 1). Based on protein subcellular localization prediction, *NbSOD* was determined to be located in the mitochondria, while *NbPrx*, *NbCAT*, and *NbGPX* were predicted to be localized in the cytoplasm (Table 1). None of the four genes were found to contain signaling peptides (Figure S3). Our alignment sequencing analysis revealed two conserved motifs (Mn/Fe-N and Mn/Fe-C) and four Mn^2+^ binding sites (His^43^, His^91^, Asp^177^, and His^181^) in *NbSOD*, three conserved motifs (APC-TSA, Redoxin, and 1-cysprx-c) and a single conserved Cys residue at position 46 in *NbPrx*, two conserved motifs (Catalase and Catalase-rel) in *NbCAT*, and two conserved motifs (Ahpc-TSA and GsHPX) in *NbGPX* (Figure 3). Through bioinformatics analysis, *NbSOD* was identified as MnSOD, belonging to the Fe/Mn SOD family; *NbPrx* was classified as 1-Cys peroxiredoxins of the peroxidase family(Prx6); *NbCAT* was recognized as a classical CAT; and *NbGPX* was determined to be cytoplasmic GPX (GPX1).

**Figure 4:**
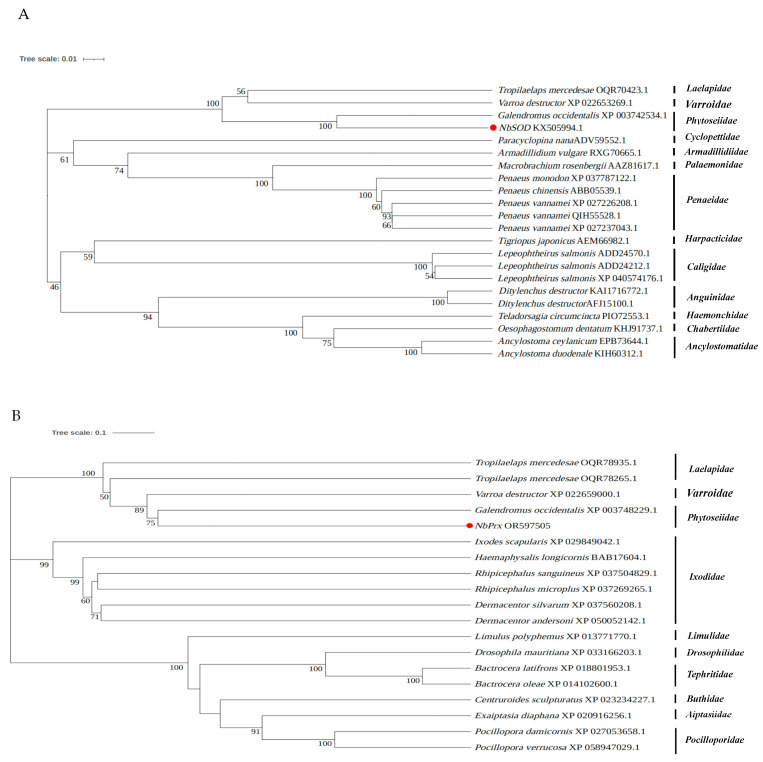

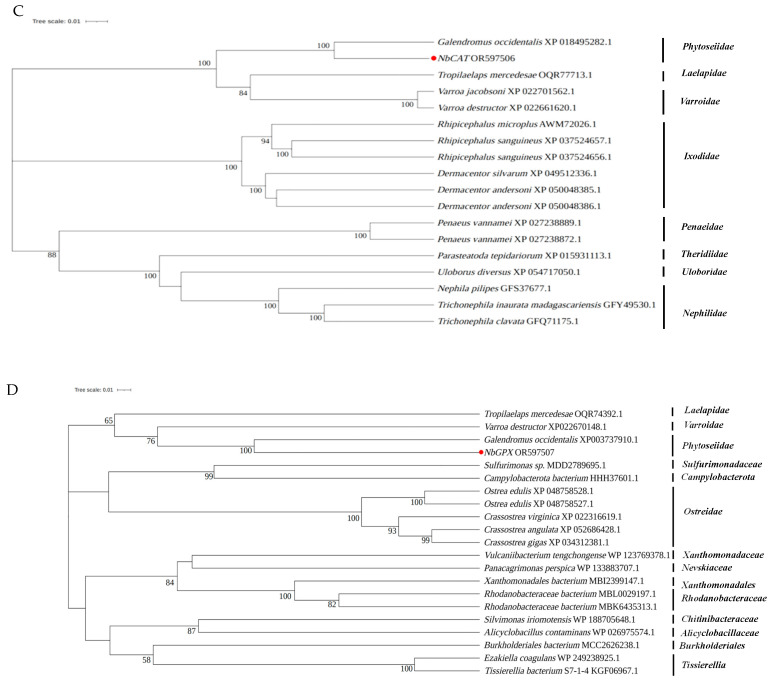
Phylogenetic relationships of the newly identified antioxidant enzyme genes ((**A**) *NbSOD*, (**B**) *NbPrx*, (**C**) *NbCAT*, and (**D**) *NbGPX*) in *N. barkeri* and others from different species. The *NbSOD*, *NbPrx*, *NbCAT*, and *NbGPX* are marked with red dots.

Upon sequences comparison, it was found that *NbSOD* from *N. barkeri* shared a sequence identity of 75.76–93.95% with other species, whereas *NbPrx* and *NbCAT* exhibited a sequence identity of 81.57–96.30% and 73.78–93.60%, respectively. *NbGPX* showed a sequence identity of 66.88–68.79% with other species. Notably, *Galendromus occidentalis* exhibited the highest similarity and sequence identity with *NbSOD*, *NbPrx*, and *NbCAT*, with sequence identity reaching 93.95%, 97.29%, and 93.60%, respectively (Table S5). Similarly, *NbGPX* shared the highest similarity and sequence identity with *Crassostrea gigas*, reaching 68.79% (Table S5). Furthermore, the phylogenetic tree analysis revealed that *NbSOD*, *NbPrx*, *NbCAT,* and *NbGPX* were the closest evolutionary relatives to *Galendromus occidentalis* (Figure 4).

### 3.4. Transcriptional Expression of Antioxidant Enzyme Genes under Different Heat Stress Conditions

**Figure 5:**
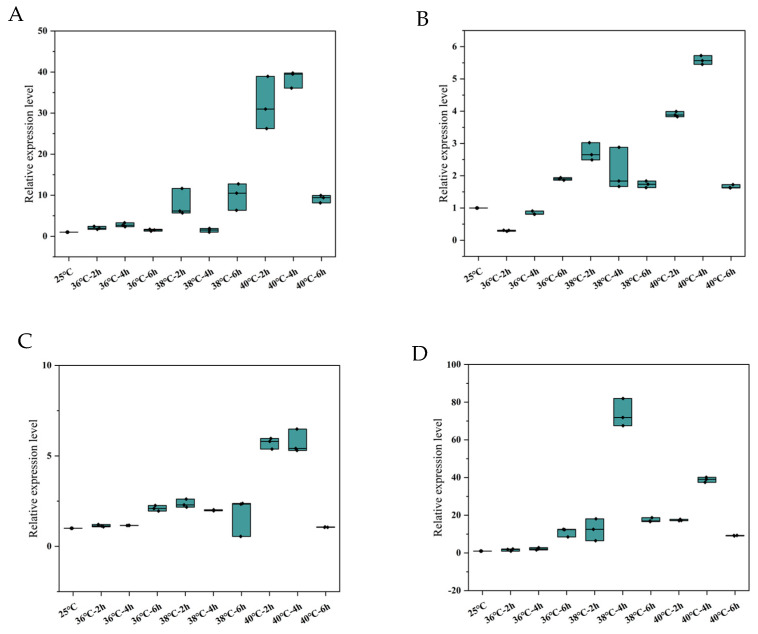
Relative expression levels of the four differentially expressed antioxidant enzyme genes ((**A**). *NbSOD*, (**B**). *NbPrx*, (**C**). *NbCAT* and (**D**). *NbGPX*) in adult female *N. barkeri*. “——”: median; “·”: individual value.

The expression patterns of these genes in *N. barkeri* were found to have differed after short-term high-temperature stress. Interestingly, at 2 h stress time, the highest expression levels of *NbSOD*, *NbPrx*, *NbCAT*, and *NbGPX* genes were observed at 40 °C followed by 38 °C. On the other hand, at 36 °C, the transcriptional expressions of *NbSOD*, *NbPrx*, and *NbGPX* were not significantly different from those of the control, while *NbCAT* was significantly lower. At 4 h stress time, the highest expression levels of *NbSOD*, *NbPrx*, and *NbCAT* were observed at 40 °C followed by 38 °C, while the expression of *NbGPX* was highest at 38 °C and decreased at 40 °C. At 36 °C, the transcriptional expression of *NbPrx*, *NbCAT*, and *NbGPX* was not significantly different from that of the control group, while the expression of NBSOD was significantly higher. At 6 h stress time, the transcription levels of *NbPrx* and *NbCAT* were highest at 36 °C, followed by 38 °C, while the transcription levels of *NbSOD* and *NbGPX* were highest at 38 °C, followed by 40 °C (Figure 5). The original data of the results are in Tables S6–S9.

## 4. Discussion

Temperature is the primary abiotic factor that significantly impacts the survival of organisms, particularly those that are poikilothermic and ectothermic [33]. *N. barkeri* frequently encounters high-temperature stress in both natural environments and facility agriculture. Heat stress is a significant factor that disrupts the balance of ROS, resulting in oxidative damage [27]. Antioxidant enzymes play a crucial role in efficiently eliminating ROS and mitigating their harmful effects on organisms [26,27].

The exposure of *N. barkeri* to short-term high temperatures significantly affected the activities of antioxidant enzymes. These effects were further enhanced with increasing temperature and longer exposure duration [27]. The transcriptome data revealed differential expression of four antioxidant enzyme genes (*NbSOD*, *NbPrx*, *NbCAT*, and *NbGPX*) following short-term heat stress, with all four genes showing up-regulation in their expression levels. The up-regulated expression of these four genes indicates that *N. barkeri* experiences oxidative stress when exposed to short-term high-temperature stress. This up-regulation indirectly enhances the activities of SOD, Prx, CAT, and GPX, thereby providing protection against the harmful effects of ROS on organisms.

Based on the analysis of multiple sequence alignment, *NbSOD* is categorized as a member of the Fe-Mn SOD family based on the presence of two domains specific to the Fe-Mn SOD family: Mn/Fe-N and Mn/Fe-C (Figure 3A). Due to the presence of iron sod (FeSOD) in prokaryotes and plants [34,35] and manganese sod (MnSOD) in eukaryotes [36], *NbSOD* is classified as MnSOD. *NbPrx* was classified as one of the 1-Cys peroxiredoxins due to the presence of a single conserved Cys residue at position 47 (Figure 3B) [37,38]. Peroxiredoxins (Prxs) are a highly conserved family of peroxidases that efficiently reduce peroxides. They contain a conserved cysteine residue known as the “peroxidatic” Cys, which is the site of oxidation by peroxides [39,40,41]. *NbCAT* has been identified as a typical catalase (Figure 3C) and is the most widely present and widely studied monofunctional, heme-containing enzyme in nature [42,43], which also binds NADPH as a second redox-active cofactor. They form tetramers, and in eukaryotic cells, catalases are located in peroxisomes [44,45]. Through protein subcellular localization prediction, *NbGPX* was identified to be situated in the cytoplasm (Tab1), utilizing GSH as its active center (Figure 3D). Consequently, *NbGPX* is classified as GPX1 within the GPX family [46]. GPX1, a tetrameric enzyme, is capable of reacting with H_2_O_2_ and soluble low-molecular hydroperoxides, but does not react with more intricate lipid hydroperoxides [47].

The organism’s response to oxidative stress begins with an increase in the activity of SOD [48]. There are three types of SODs in organisms: copper/zinc SODs (Cu/ZnSOD), manganese SODs (MnSOD), and iron SODs (FeSOD) [49,50,51]. In this study, *NbSOD* was identified as MnSOD (Figure 3A). MnSOD is predominantly localized within the mitochondria of eukaryotic cells [52,53], which aligns with the findings of protein subcellular localization prediction (Table 1). Mitochondria are responsible for producing energy in the cell and are also known to be a major source of ROS [54]. High temperatures accelerate the energy consumption of *N. barkeri* [55], leading to an increased demand for energy production by the mitochondria. As a result, reactive oxygen species (ROS) are generated as by-products of the mitochondrial electron transport chain [21]. Based on this, we hypothesize that the rise in ROS levels in *N. barkeri* following short-term heat stress is attributed to mitochondrial activity. SOD scavenges free radicals and converts them into H_2_O_2_, while Prx and CAT break down H_2_O_2_ [27]. Additionally, GPX commonly employs glutathione (GSH) as a reducing agent to catalyze the conversion of H_2_O_2_ or organic hydrogen peroxide into H_2_O or corresponding alcohols, respectively [56,57,58]. Based on protein subcellular localization prediction (Table 1), *NbPrx*, *NbCAT,* and *NbGPX* are all present in the cytoplasm. Following brief heat stress, *N. barkeri* generates a significant amount of ROS within the mitochondria, causing an imbalance in ROS levels within the organism. Subsequently, *NbSOD* converts ROS into H_2_O_2_ within the mitochondria, and this H_2_O_2_ is subsequently transported to the cytoplasm. In the cytoplasm, *NbPrx*, *NbCAT*, and *NbGPX* each perform distinct mechanisms to break down H_2_O_2_ into H_2_O and other harmless substances. This process effectively reduces their harmful effects, safeguarding the integrity and functionality of the cell membrane against oxidative interference and damage.

The transcriptional expression of antioxidant enzyme genes in *N. barkeri* varies in response to different external stresses. After exposure to UV-B radiation stress, the expression of three SOD genes (*Cu/ZnSOD1*, *Cu/ZnSOD2*, and *MnSOD*) and two phospholipid hydroperoxide GPX genes (*PHGPX1* and *PHGPX2*) in *N. barkeri* was up-regulated [59]. A high-temperature adapted strain (HTAS), the predatory mite *N. barkeri* was artificially selected via long-term heat acclimation (35 °C) and frequent heat hardenings. Compared to susceptible strains, the HTAS showed increased expression of 2 SOD genes and decreased expression of 3 SOD genes. Additionally, it exhibited increased expression of 6 Prx genes and 1 CAT gene, as well as decreased expression of 3 CAT genes [55]. After exposure to Fenpropathrin stress, two GPX genes exhibited up-regulation, whereas only one GPX gene showed up-regulation following pyridazine stress [26]. In addition to the variation in the number of genes that regulate the activity of antioxidant enzymes, *N. barkeri* also exhibited differences in the types of antioxidant enzyme genes in response to external stress. Through a comparison of the four antioxidant genes in Blastp, the *NbPrx*, *NbCAT*, and *NbGPX* were not identified, while the *NbSOD was* previously reported by Tian et al. [59].

The expression analysis revealed significant differences in the four antioxidant genes in *N. barkeri* after short-term high-temperature exposure. Similarly, the highest expression levels of all four genes were observed at 40 °C for 4 h, except for *NbGPX*, which exhibited its maximum expression value at 38 °C for 4 h. These findings are consistent with similar results obtained in other organisms, such as green peach aphid *Myzus persicae* [60], *Drosophila* [61], and *Mytilus galloprovincialis* [62]. It suggests that the up-regulation of antioxidant genes may occur within a specific temperature range, as extreme temperatures can disrupt cell redox homeostasis, carbohydrate and energy metabolism [63], and cause damage to cytoskeletal structural elements [64]. Furthermore, the negative effects of extreme temperatures on gene expression become more pronounced with longer exposure durations [65].

## 5. Conclusions

In this study, we screened and identified four up-regulated antioxidant enzyme genes in *N. barkeri* following exposure to heat stress conditions. The results showed that *NbSOD*, *NbPrx*, *NbCAT*, and *NbGPX* played vital role in responding to short-term high-temperature stress. The significant up-regulation of transcriptional expression in antioxidant enzyme genes serves as an indication of oxidative stress within the organism. However, further investigation is required to determine if this phenomenon also occurs in other organisms.

## Figures and Tables

**Table 1:** Detailed information of antioxidant enzyme genes from *N. barkeri*.

Gene	ORF	aa	Formula	Molecular Weight (kDa)	Theoretical pI	Protein Subcellular Localization Prediction
*NbSOD*	648	215	C_1066_H_1656_N_294_O_310_S_5_	23.711	8.85	Mitochondrial
*NbPrx*	675	224	C_1124_H_1769_N_291_O_325_S_14_	25.000	5.87	Cytoplasmic
*NbCAT*	1515	504	C_2557_H_3880_N_708_O_756_S_18_	57.212	7.22	Cytoplasmic
*NbGPX*	498	265	C_850_H_1302_N_208_O_247_S_5_	18.547	5.50	Cytoplasmic

## Data Availability

All data support for this research is included in this article and supplementary file.

## Supplementary Materials

The following supporting information can be downloaded at: https://www.mdpi.com/article/10.3390/antiox12111998/s1.
