# Supplementary material for: Selection, Identification, and Transcript Expression Analysis of Antioxidant Enzyme Genes in Neoseiulus barkeri after Short-Term Heat Stress
Source: Antioxidants (Basel). 2023 Nov 13;12(11):1998. doi: 10.3390/antiox12111998 (PMC10669032; doi:10.3390/antiox12111998)
Supplement: Supplementary file 1 [file antioxidants-12-01998-s001.zip › Figure S1-S3.pdf]

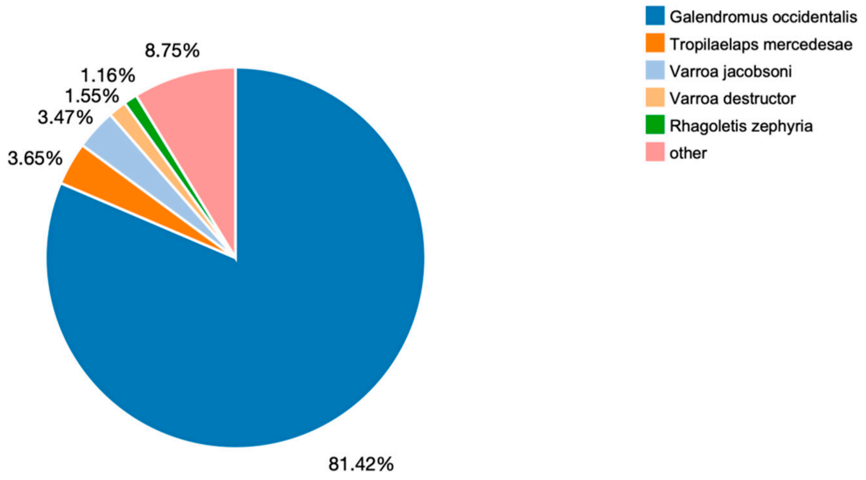

Figure S1. NR species annotation distribution. According to the NR annotation results, the proportion of different species on the annotation was counted, and the species distribution map was drawn. Different species are displayed in different colors.

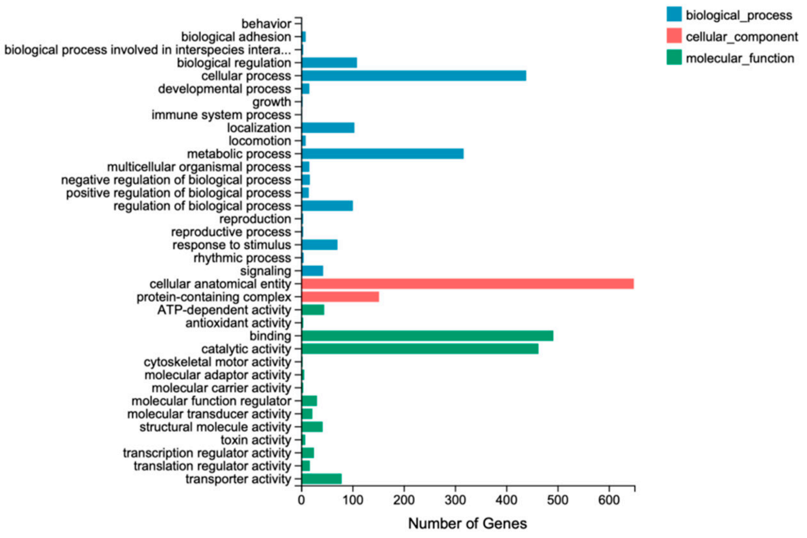

Figure S2. Differential gene GO classification. Gene Ontology is divided into three functional classes: molecular function, cellular component, and biological process. Functional classification was performed according to the differential gene test results. There are various levels of subcategories under each category.

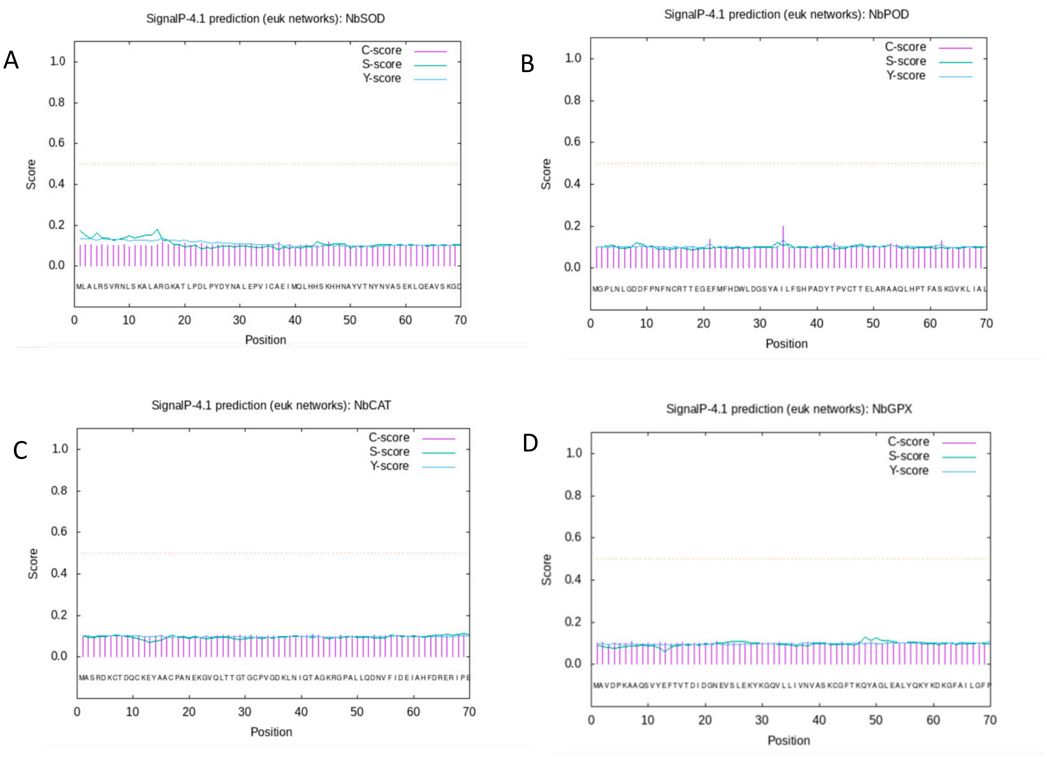

Figure S3。signaling peptides of A.*NbSOD*, *NbPOD*, *NbCAT*, and *NbGPX*. None of the four genes were found to contain signaling peptides.
