# Supplementary material for: Selection, Identification, and Transcript Expression Analysis of Antioxidant Enzyme Genes in Neoseiulus barkeri after Short-Term Heat Stress
Source: Antioxidants (Basel). 2023 Nov 13;12(11):1998. doi: 10.3390/antiox12111998 (PMC10669032; doi:10.3390/antiox12111998)
Supplement: Supplementary file 1 [file antioxidants-12-01998-s001.zip › Table S1.pdf]

Table S1. The primers used for cloning CDS sequences of four antioxidant genes

Gene ID	Primer Name	Sequence (5'-3')
KX505994.1	SOD-F	AACTGTCGGCATCGGAGTTC
KX505994.1	SOD-R	GCTAAAATGTGACGCCGGAA
OR597505	POD-F	ATGGGACCGCTGAATCTTGG
OR597505	POD-R	TTAGTCCATGGTGGTACGCAT
OR597506	CAT-F	TACGTTCGCGTCATCGACTT
OR597506	CAT-R	TTCTCACCAACAGTGGGAGC
OR597507	GPX-F	TTCGCTGTAACGATGGCTGT
OR597507	GPX-R	TCGCGTTTTCCCTCGTGTAA
