# Supplementary material for: Selection, Identification, and Transcript Expression Analysis of Antioxidant Enzyme Genes in Neoseiulus barkeri after Short-Term Heat Stress
Source: Antioxidants (Basel). 2023 Nov 13;12(11):1998. doi: 10.3390/antiox12111998 (PMC10669032; doi:10.3390/antiox12111998)
Supplement: Supplementary file 1 [file antioxidants-12-01998-s001.zip › Table S2.pdf]

Table S2. The primers used for RT-qPCR of four antioxidant genes.

Gene ID	Primer Name	Sequence (5'-3')
KX505994.1	SOD-F	CTCTCTAAAGCCCTCGCTCG
KX505994.1	SOD-R	GCGATCTGTGCGTTGACATC
OR597505	CAT-F	ACTTCGCTGAGGTGGAACAG
OR597505	CAT-R	GAACAGACGTCCTTGGAGCA
OR597506	POD-F	GGACCGCTGAATCTTGGTGA
OR597506	POD-R	TGGAACATGAACTCGCCCTC
OR597507	GPX-F	TCAAGGAGTTCTGCTCGCTG
OR597507	GPX-R	TGTCGACGAGGAACTTGGTG
