# Supplementary material for: Selection, Identification, and Transcript Expression Analysis of Antioxidant Enzyme Genes in Neoseiulus barkeri after Short-Term Heat Stress
Source: Antioxidants (Basel). 2023 Nov 13;12(11):1998. doi: 10.3390/antiox12111998 (PMC10669032; doi:10.3390/antiox12111998)
Supplement: Supplementary file 1 [file antioxidants-12-01998-s001.zip › Table S4.pdf]

Table S4 The Unigene annotation

Values	Number	Percentage
Total	37,379	100%
NR	21,954	58.73%
NT	8,951	23.95%
Swissprot	13,462	36.01%
KEGG	16,044	42.92%
KOG	12,968	34.69%
Pfam	15,042	40.24%
GO	11,039	29.53%
Intersection	3,046	8.15%
Overall	23,893	63.92%
