# Supplementary material for: Selection, Identification, and Transcript Expression Analysis of Antioxidant Enzyme Genes in Neoseiulus barkeri after Short-Term Heat Stress
Source: Antioxidants (Basel). 2023 Nov 13;12(11):1998. doi: 10.3390/antiox12111998 (PMC10669032; doi:10.3390/antiox12111998)
Supplement: Supplementary file 1 [file antioxidants-12-01998-s001.zip › Table S5.pdf]

Table S4. The analysis of the similarity and identity of four antioxidant genes with other species.

Gene name	Species	Accession number	Similarity (%)	Identity (%)
NbSOD	*Galendromus occidentalis*	XP_003742534.1	99%	93.95%
	Varroa destructor	XP_022653269.1	99%	81.78%
	Tropilaelaps mercedesae	OQR70423.1	99%	79.53%
	Armadillidium vulgare	RXG70665.1	91%	76.14%
	Penaeus chinensis	XP_047497250.1	91%	76.14%
	Cryptotermes secundus		91%	75.76%
NbPOD	*Galendromus occidentalis*	XP_003748229.1	96%	96.30%
	Varroa destructor	XP_022659000.1	95%	
	Tropilaelaps mercedesae	OQR78265.1	96%	87.96%
		ABR23497.1	82%	84.41%
		XP_029849042.1	96%	83.33%
		BAB17604.1	96%	81.57%
NbCAT	*Galendromus occidentalis*	XP_018495283.1	99%	93.60%
	Tropilaelaps mercedesae	OQR77713.1	99%	86.06%
	Varroa destructor	XP_022661621.1	99%	84.20%
	Eriocheir sinensis	XP_050722477.1	93%	74.42%
	Nephila pilipes	GFS37677.1	98%	73.99%
	Trichonephila clavata	GFQ71175.1	97%	73.78%
NbGPX	*Crassostrea gigas*	XP_034312380.1	93%	68.79%
	Crassostrea angulata	XP_052686427.1	93%	68.79%
	Ostrea edulis	XP_048758528.1	95%	68.55%
	Burkholderiales bacterium	MCC2626238.1	95%	68.35%
	Crassostrea virginica	XP_022316619.1	95%	67.30%
	Panacagrimonas perspica	WP_133883707.1	94%	66.88%
